# Prevalence and Characterization of CRISPR Locus 2.1 Spacers in Escherichia coli Isolates Obtained from Feces of Animals and Humans

**DOI:** 10.1128/spectrum.04934-22

**Published:** 2023-01-31

**Authors:** Hanseob Shin, Yongjin Kim, Tatsuya Unno, Hor-Gil Hur

**Affiliations:** a School of Earth Sciences and Environmental Engineering, Gwangju Institute of Science and Technology (GIST), Gwangju, Republic of Korea; b Faculty of Biotechnology, College of Applied Life Sciences, SARI, Jeju National University, Jeju, Republic of Korea; University of Minnesota Twin Cities

**Keywords:** clustered regularly interspaced short palindromic repeat, spacer, *Escherichia coli*, bacteriophage, microbial source tracking, CRISPR, *Caudovirales*

## Abstract

The clustered regularly interspaced short palindromic repeat (CRISPR) has been studied as an immune system in prokaryotes for the survival of bacteriophages. The CRISPR system in prokaryotes records the invasion of bacteriophages or other genetic materials in CRISPR loci. Accordingly, CRISPR loci can reveal a history of infection records of bacteriophages and other genetic materials. Therefore, identification of the CRISPR array may help trace the events that bacteria have undergone. In this study, we characterized and identified the spacers of the CRISPR loci in Escherichia coli isolates obtained from the feces of animals and humans. Most CRISPR spacers were found to stem from phages. Although we did not find any patterns in CRISPR spacers according to sources, our results showed that phage-derived spacers mainly originated from the families *Inoviridae*, *Myoviridae*, *Podoviridae*, and *Siphoviridae* and the order *Caudovirales*, whereas plasmid-derived CRISPR spacers were mainly from the *Enterobacteriaceae* family. In addition, it is worth noting that the isolates from each animal and human source harbored source-specific spacers. Considering that some of these taxa are likely found in the gut of mammalian animals, CRISPR spacers identified in these E. coli isolates were likely derived from the bacteriophageome and microbiome in closed gut environments. Although the bacteriophageome database limits the characterization of CRISPR arrays, the present study showed that some spacers were specifically found in both animal and human sources. Thus, this finding may suggest the possible use of E. coli CRISPR spacers as a microbial source tracking tool.

**IMPORTANCE** We characterized spacers of CRISPR locus 2.1 in E. coli isolates obtained from the feces of various sources. Phage-derived CRISPR spacers are mainly acquired from the order *Caudovirales,* and plasmid-derived CRISPR spacers are mostly from the *Enterobacteriaceae* family. This is thought to reflect the microbiome and phageome of the gut environment of the sources. Hence, spacers may help track the encounter of bacterial cells with bacterial cells, viruses, or other genetic materials. Interestingly, source-specific spacers are also observed. The identification of source-specific spacers is thought to help develop the methodology of microbial source tracking and understanding the interactions between viruses and bacteria. However, very few spacers have been uncovered to track where they originate. The accumulation of genome sequences can help identify the hosts of spacers and can be applied for microbial source tracking.

## INTRODUCTION

Escherichia coli is a Gram-negative bacterium belonging to the *Enterobacteriaceae* family and is known to mainly inhabit the intestines of warm-blooded animals. E. coli is usually a commensal but occasionally presents as an opportunistic pathogen that causes diarrheic disease in animals (1). The E. coli population structure in the gut of animals can be influenced by several host factors, such as genetics, dietary habits, and age (2). The ecology of bacteriophages in the gut also affects the structure of E. coli populations in the gut (3). It should be noted that bacteriophages account for a large portion of the gut microbiome (~10^15^ bacteriophages, known as the phageome), which remains poorly understood in the gut microbiome (4).

With the development of metagenomics, an increasing amount of bacteriophage genomic sequence data has been stored at the National Center for Biotechnology Information (NCBI). However, due to limitations in evaluating biological properties, the systematic classification of these bacteriophages is often incomplete (5). Of more than 8,000 complete genome sequences, more than 90% were unidentified taxonomies based on metagenomic data (6). More than half of the sequences belonged to the *Siphoviridae* family, followed by *Myoviridae* (17%) and *Podoviridae* (12%). A diversity of bacteriophages has been examined in the soil (7), aquatic (8), and human gut environments (9). In the marine environment, nontailed phages were the most abundant, followed by tailed phages of *Myoviridae* (14%), *Podoviridae* (6%), and *Siphoviridae* (1%) in the order *Caudovirales*, which was also confirmed by metagenomic data. In contrast, tailed phages were dominant in the soil samples examined. Most belonged to the order *Caudovirales*. The phage community of the human gut is mainly composed of members of the order *Caudovirales*, with the majority of unclassified phage groups.

Coliphages, which are generally present in the gut of humans and other warm-blooded animals, are grouped into somatic and F-specific coliphages based on the morphology of subsections to infect coliform bacteria, such as E. coli and Salmonella enterica serovar Typhimurium (10). Phages in human guts are generally temperate (11), and thus, temperate phage-bacterial strain interactions may occur. The interaction between coliphages and coliform bacteria affects the microbial ecology and evolves the bacterial community through horizontal gene transfer, thereby influencing bacterial diversity. For example, the properties of E. coli can be changed by bacteriophages from commensal to pathogenic under the selective pressure of macrophages (12). In addition, coliphages can be applied to treat infections caused by pathogenic E. coli because they have high specificity to the host bacteria without affecting other bacteria (13).

Coliphages have also been suggested as indicators of fecal contamination in aquatic environments because of the shortcomings of fecal indicator bacteria, such as their persistence, size, and growth in a specific environment (14). In addition, coliphages have been suggested as markers for source tracking of anthropogenic activity (15). Indeed, genetic analysis of F^+^ RNA coliphages from various fecal samples of humans and animals could verify their usability as a tool for microbial source tracking (MST) (16).

Several tools, such as fingerprinting (17), ribotyping (18), and sequence typing (19), have been applied for source tracking of E. coli from various sources. The clustered regularly interspaced short palindromic repeat (CRISPR) system showed a pattern similar to that of multilocus sequence typing (MLST) (20), suggesting it as a potential tool for source tracking. Environmental E. coli has a CRISPR system (21) which consists of an array of CRISPR loci and a series of CRISPR-associated sequence (Cas) genes (22). The CRISPR system is mediated by three stages arrayed by recorded spacers, in which the new spacer is located on the nearest side of the leader sequence of the CRISPR array (23). It can provide a series of events in chronological order that the bacteria have faced against foreign genetic material (24). The CRISPR system of E. coli contains subtypes I-E and I-F (25). The I-E and I-F types include CRISPR 1 and CRISPR 2 (CRISPR loci 2.1, 2.2, and 2.3), and CRISPR 3 and CRISPR 4, respectively. The diversity of CRISPR locus 2.1 and CRISPR I-F types is highly involved in the acquisition of spacers due to the high diversity of those subsets (26). The CRISPR I-F type cannot incorporate spacers from RNA-based viruses because of the absence of reverse transcriptase (27). In addition, although CRISPR I-F type was suggested to have the potential for typing the B2 group of E. coli (28), none of E. coli isolates in this study belonged to the phylogenetic B2 group (29). For these reasons, CRISPR locus 2.1 was targeted for characterization in this study.

Here, we hypothesized that we could identify sources of E. coli through characterization and identification of spacers of CRISPR locus 2.1. In this study, spacers of CRISPR locus 2.1 in 141 E. coli isolates obtained from humans and animals were characterized to demonstrate the usability of CRISPR spacers for source tracking.

## RESULTS

### CRISPR locus 2.1 of E. coli isolates.

Of the 569 E. coli isolates, 141 harbored CRISPR locus 2.1. The highest number of isolates carrying CRISPR locus 2.1 was found in ducks (*n *=* *53), followed by pigs (*n *=* *43), humans (*n *=* *19), beef cows (*n *=* *8), milk cows (*n *=* *8), patients (*n *=* *8), and chickens (*n *=* *2) (Table 1). The carriers of E. coli carrying CRISPR locus 2.1 were patients (38.1%), pigs (35.0%), ducks (31.5%), humans (20.7%), dairy cows (14.8%), beef cows (13.8%), and chickens (3.8%), respectively. The highest number of spacers was found in ducks (*n* = 210), followed by pigs (*n *=* *160), humans (*n *=* *118), beef cows (*n *=* *58), milk cows (*n *=* *54), patients (*n *=* *23), and chickens (*n *=* *14). In addition, source-specific spacers were present in CRISPR locus 2.1 from ducks (*n *=* *88), humans (*n *=* *58), pigs (*n *=* *53), dairy cows (*n *=* *26), beef cows (*n *=* *20), and patients (*n *=* *2), while no source-specific spacers were found in chickens in this study.

**TABLE 1:** Detection of CRISPR locus 2.1 and spacers from E. coli isolates

Source	Total no. of E. coli isolates	No. (%) of strains with CRISPR	No. of spacers	No. of host-specific spacers
Humans	92	19 (20.7)	118	58
Patients	21	8 (38.1)	23	2
Pigs	123	43 (35.0)	164	53
Chickens	53	2 (3.8)	14	0
Ducks	168	53 (31.5)	210	88
Beef cows	58	8 (13.8)	58	20
Dairy cows	54	8 (14.8)	54	26
Total	569	141 (24.8)	641	247

### Identification of protospacers from CRISPR systems of E. coli isolates.

**FIG 1:**
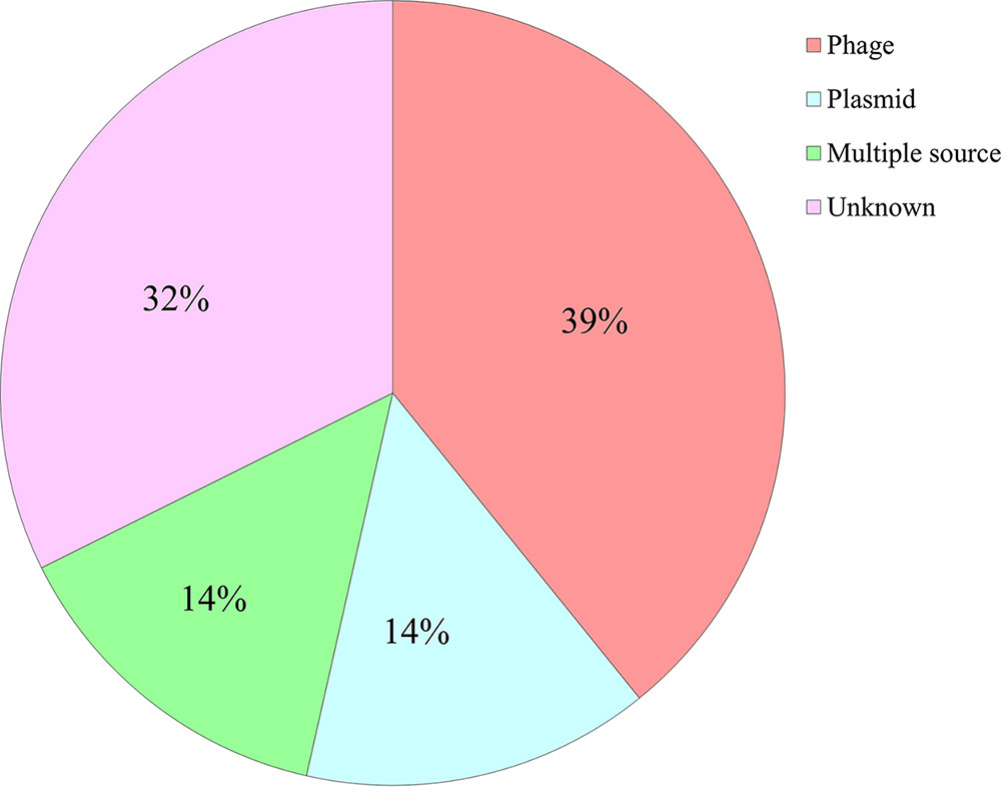
Composition of hosts of spacers recorded in the CRISPR 2.1 locus of E. coli isolates.

Identification of spacers of CRISPR locus 2.1 revealed that the majority of these spacers originated from phages (39%) and plasmids (14%), while 32% and 14% originated from unknown and multiple sources, respectively (Fig. 1). Host-identified spacers were most frequently found in ducks (*n *=* *381), followed by pigs (*n *=* *185), humans (*n *=* *81), beef cows (*n *=* *55), dairy cows (*n* = 3 2), patients (*n *=* *16), and chickens (*n *=* *11) (Table 2). Except for the “unidentified family,” the *Myoviridae* family was most frequently found (*n* = 1 to 46 [9.1 to 43.8% of the total]), while spacers from unknown phages ranged from 3 to 69 (0 to 21%). Spacers from the *Siphoviridae* and *Podoviridae* families were also frequently detected (*n *=* *0 to 28 [0 to 9.4%] and *n *=* *0 to 11 [0 to 6.2%], respectively). A few spacers originating from the *Inoviridae* family (*n *=* *2 [0.5%]), Salmonella phage S137 (*n *=* *2 [0.5%]), and Salmonella phage SPN3UB (*n *=* *1 [0.3%]) were detected only in the ducks. On the other hand, the majority of plasmid-derived spacers were from E. coli strain LD91-1 plasmid pLD91-1-76kb (*n *=* *2 to 42 [6.3 to 45.5%]), followed by E. coli strain IOMTU792 plasmid pIOMTU792 (*n *=* *0 to 40 [0 to 12.5%]), Escherichia albertii strain sample 167 plasmid pESA138_1 (*n *=* *0 to 17 [0 to 4.5%]), an E. coli strain 2012C-4221 plasmid (*n *=* *0 to 12 [0 to 3.8%]), and a *Pantoea* sp. strain CCBC3-3-1 plasmid (*n *=* *0 to 22 [0 to 6.3%]). A few spacers originating from the Phaeobacter piscinae strain P13 plasmid pP13_a (*n *=* *0 to 1 [0 to 0.3%]), Salmonella enterica subsp. strain SA20063285 plasmid pIncI1.1 (*n *=* *0 to 4 [0 to 2.2%]), Sinorhizobium meliloti RU11001 plasmid pSymB (*n *=* *0 to 2 [0 to 2.5%]), and Vibrio parahaemolyticus FORC_014 plasmid pFORC14 (*n *=* *0 to 6 [0 to 10.9%]).

**TABLE 2:** Detection of CRISPR locus 2.1 and spacers from E. coli isolates

Phage or plasmid	Host identified	No. (%) of host-identified spacers from:						
		Humans	Patients	Pigs	Chickens	Ducks	Beef cows	Dairy cows
Phages	Unidentified family of *Caudovirales* order	23 (28.4)	3 (18.8)	46 (24.9)	3 (27.3)	69 (18.1)	16 (29.1)	8 (25)
	*Inoviridae* phage	0 (0)	0 (0)	0 (0)	0 (0)	2 (0.5)	0 (0)	0 (0)
	*Myoviridae* phage	9 (11.1)	7 (43.8)	34 (18.4)	1 (9.1)	46 (12.1)	8 (14.5)	11 (34.4)
	*Podoviridae Uetakevirus* phage	5 (6.2)	0 (0)	9 (4.9)	0 (0)	11 (2.9)	0 (0)	0 (0)
	Salmonella phage S137	0 (0)	0 (0)	0 (0)	0 (0)	2 (0.5)	0 (0)	0 (0)
	Salmonella phage SPN3UB	0 (0)	0 (0)	0 (0)	0 (0)	1 (0.3)	0 (0)	0 (0)
	*Siphoviridae* phage	5 (6.2)	1 (6.3)	9 (4.9)	0 (0)	28 (7.3)	4 (7.3)	3 (9.4)
	Unknown phage	14 (17.3)	0 (0)	31 (16.8)	2 (18.2)	80 (21)	11 (20)	3 (9.4)

Bacterial plasmids	Escherichia albertii strain sample 167 plasmid pESA138_1	1 (1.2)	0 (0)	7 (3.8)	0 (0)	17 (4.5)	2 (3.6)	0 (0)
	Escherichia coli strain 2012C-4221 plasmid	1 (1.2)	0 (0)	7 (3.8)	0 (0)	12 (3.1)	0 (0)	0 (0)
	Escherichia coli strain LD91-1 plasmid pLD91-1-76kb	15 (18.5)	2 (12.5)	12 (6.5)	5 (45.5)	42 (11)	7 (12.7)	2 (6.3)
	Escherichia coli strain IOMTU792 plasmid pIOMTU792	3 (3.7)	2 (12.5)	13 (7)	0 (0)	40 (10.5)	0 (0)	3 (9.4)
	*Pantoea* sp. strain CCBC3-3-1 plasmid	1 (1.2)	1 (6.3)	8 (4.3)	0 (0)	22 (5.8)	1 (1.8)	2 (6.3)
	Phaeobacter piscinae strain P13 plasmid pP13_a	0 (0)	0 (0)	0 (0)	0 (0)	1 (0.3)	0 (0)	0 (0)
	Salmonella enterica subsp. SA20063285 plasmid pIncI1.1	0 (0)	0 (0)	4 (2.2)	0 (0)	2 (0.5)	0 (0)	0 (0)
	Sinorhizobium meliloti RU11001 plasmid pSymB	2 (2.5)	0 (0)	0 (0)	0 (0)	1 (0.3)	0 (0)	0 (0)
	Vibrio parahaemolyticus FORC_014 plasmid pFORC14	2 (2.5)	0 (0)	5 (2.7)	0 (0)	5 (1.3)	6 (10.9)	0 (0)

Total		81 (100)	16 (100)	185 (100)	11 (100)	381 (100)	55 (100)	32 (100)

### Occurrence of spacers from animals and humans.

**FIG 2:**
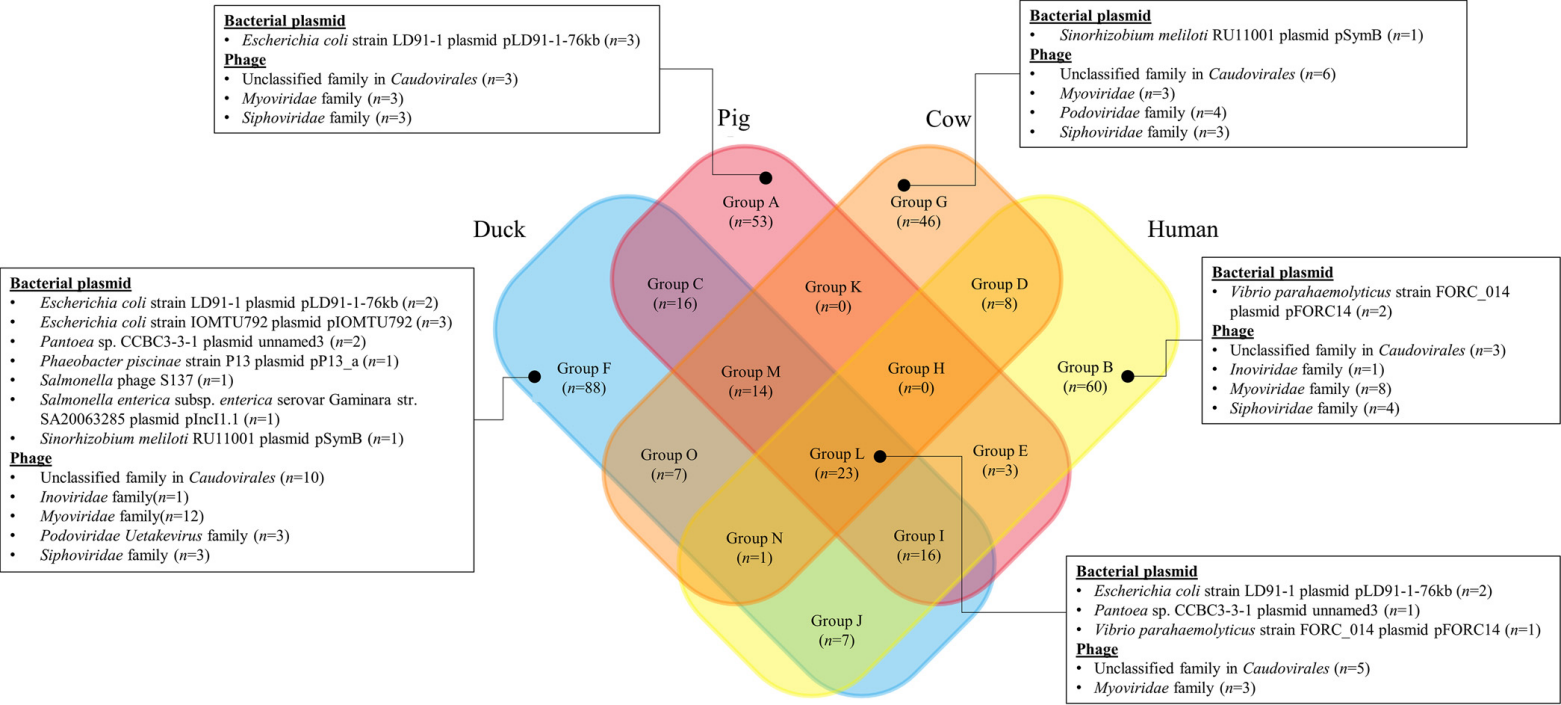
Occurrence of spacers in source animals and humans.

The occurrence of spacers in animal and human sources was also investigated (Fig. 2). Some common spacers were grouped separately due to their sequence dissimilarities. This is thought to result from the capture and processing of different sequence fragments of the same spacer from foreign DNA sequences. Spacers from *Myoviridae*, *Siphoviridae*, and unidentified families of *Caudovirales* were found in all of the source animals and humans. Spacers from the *Podoviridae* family were found in groups F and G. Duck E. coli contained the most diverse plasmid-derived bacterial spacers (group F). In addition, we did not observe the co-occurrence of spacers among pigs, cows, and humans (group H), as well as in pigs and cows (group K). Among the spacers from bacterial plasmids, E. coli strain LD91-1 plasmid pLD91-1-76kb-derived spacers commonly occurred among all sources.

**FIG 3:**
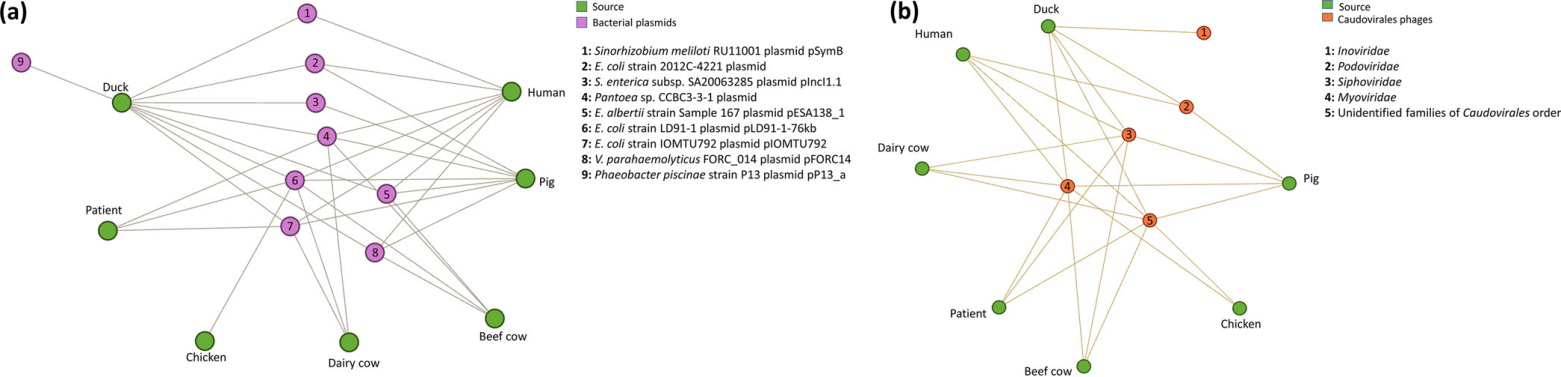
Network analysis of hosts’ plasmids (a) and phages (b) of spacers with source animals and humans.

Network analysis showed that most plasmid- and phage-originated spacers were highly associated with E. coli from animals and humans (Fig. 3). The isolates from ducks (*n *=* *8) carried spacers from bacterial plasmids (Fig. 3a), followed by pigs (*n *=* *7), humans (*n *=* *7), beef cows (*n *=* *4), dairy cows (*n *=* *3), patients (*n *=* *3), and chickens (*n *=* *3). Among the host phages (Fig. 3b), the most diverse spacers were obtained from ducks (*n *=* *5), followed by pigs (*n *=* *4), humans (*n *=* *4), beef cows (*n *=* *3), dairy cows (*n *=* *3), patients (*n *=* *3), and chickens (*n *=* *2). Isolates from ducks were found to harbor spacers from specific host plasmids and phages, such as Phaeobacter piscinae strain P13 plasmid pP13_a and phage *Inoviridae.*

**FIG 4:**
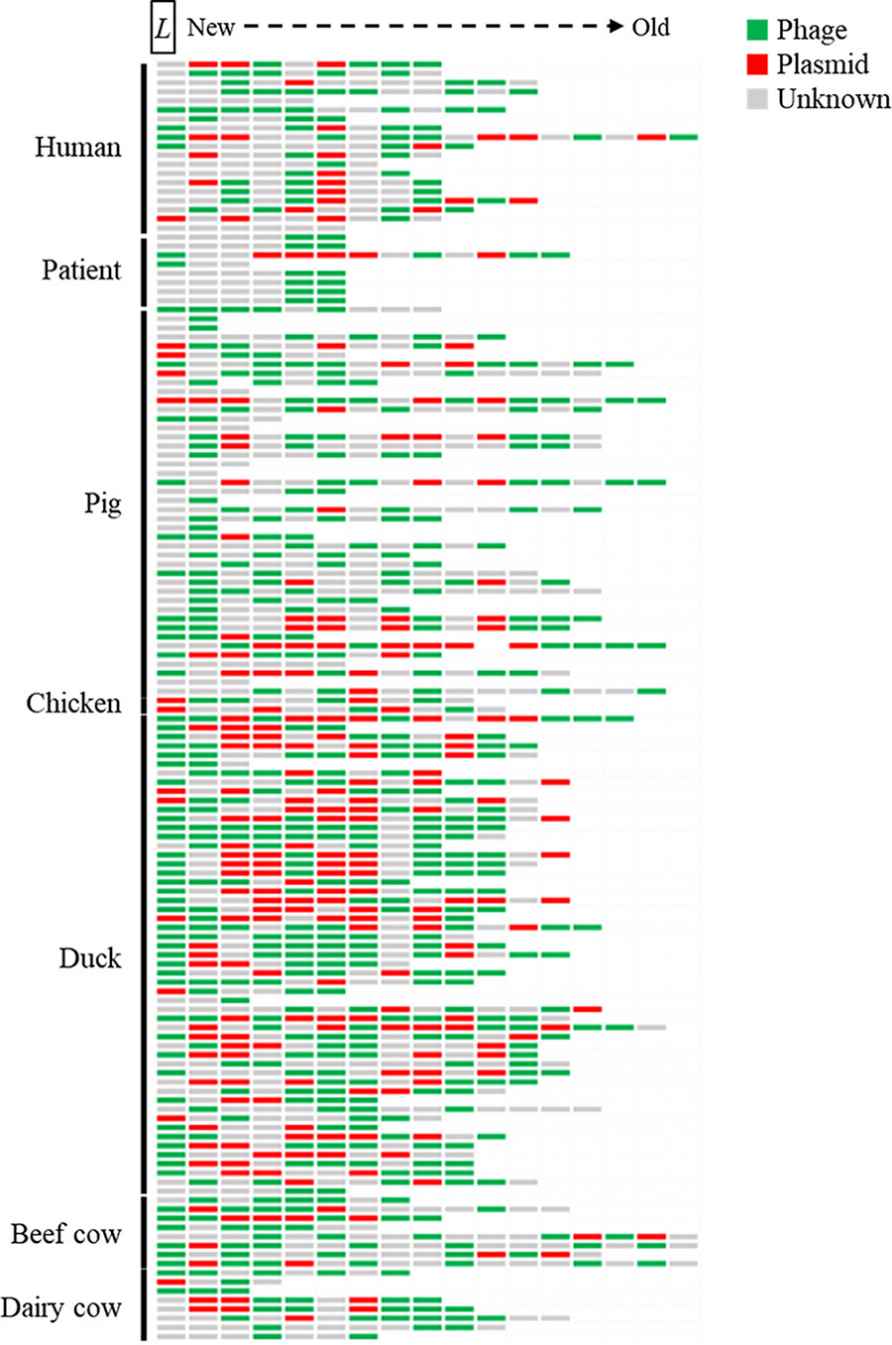
Profiles of spacers detected from each E. coli isolate from source human and animals. *L* represents the location of leader sequence. A new spacer was added downstream of the leader sequence (*L*) of the CRISPR array.

To investigate the occurrence patterns of spacers, those from each E. coli isolate were arranged according to animal and human sources (Fig. 4). E. coli isolates from beef cows and ducks seemed to encounter host phage-originated spacers more commonly. The CRISPR array of E. coli isolates from all source animals and humans contained a variable portion of the spacer derived from phages and plasmids. Phage-derived spacers were relatively abundant in the CRISPR array of E. coli isolates from beef cows, milk cows, humans, patients, and pigs. A similar portion of plasmid- and phage-originated spacers was distributed in the CRISPR array among chicken isolates. Compared to the spacers among the sources, fewer spacers from the unknown host were found in the chicken isolates.

### Detection of source animals and human-specific spacers.

The most diverse source-specific spacers were found in ducks (*n *=* *88), followed by human patients (*n *=* *58), dairy cows (*n *=* *53), beef cows (*n *=* *26), pigs (*n *=* *19), and humans (*n *=* *2) in E. coli isolates (Table 3). In beef cow and human sources, the 26 and 2 spacers, respectively, did not overlap. In ducks, D12 and D13 source-specific spacers were most frequently found at *n *=* *4, followed by D11, D32, D97, D104, and D105 (*n *=* *3), another 14 spacers (*n *=* *2), and 67 other spacers (*n *=* *1). In dairy cows, MC294 and MC296 source-specific spacers were most frequently found at *n *=* *5, followed by MC278, MC289, and MC295 (*n *=* *4), MC288 (*n *=* *3), another 10 spacers (*n *=* *2), and the other 37 spacers (*n *=* *1). In human patients, the P433 source-specific spacer was most frequently found as *n *=* *3, followed by another other 10 spacers (*n *=* *2) and the other 46 spacers (*n *=* *1). Among the pig sources, 10 pig source-specific spacers were the most frequent as *n *=* *3, followed by Pig51, Pig352, Pig353, and Pig354 (*n *=* *2) and the other five spacers (*n *=* *1).

**TABLE 3:** Source-specific spacers of strains from humans and source animals

Source (no. of spacers)	Source-specific spacers (frequency of detection)
Humans (*n *=* *2)	Human371, Human374
Patients (*n *=* *58)	P433 (3), P389 (2), P390 (2), P391 (2), P392 (2), P393 (2), P394 (2), P395 (2), P396 (2), P397 (2), P398 (2), P376, P377, P378, P379, P380, P381, P382, P383, P384, P385, P386, P387, P388, P399, P400, P401, P402, P403, P404, P405, P406, P408, P409, P410, P413, P414, P415, P416, P417, P418, P419, P420, P422, P425, P426, P427, P428, P429, P430, P431, P432, P434, P435, P436, P437, P438, P439

Pigs (*n *=* *19)	Pig44 (3), Pig45 (3), Pig46 (3), Pig47 (3), Pig48 (3), Pig50 (3), Pig52 (3), Pig53 (3), Pig55 (3), Pig56 (3), Pig51 (2), Pig352 (2), Pig353 (2), Pig354 (2), Pig49, Pig54, Pig307, Pig350, Pig355
Ducks (*n *=* *88)	D12 (4), D13 (4), D11 (3), D32 (3), D97 (3), D104 (3), D105 (3), D35 (2), D96 (2), D98 (2), D99 (2), D100 (2), D101 (2), D102 (2), D103 (2), D142 (2), D143 (2), D144 (2), D145 (2), D175 (2), D196 (2), D33, D34, D36, D37, D38, D39, D40, D41, D42, D43, D69, D71, D72, D95, D109, D110, D111, D112, D113, D118, D132, D133, D134, D135, D136, D137, D151, D156, D157, D158, D173, D177, D179, D180, D181, D182, D186, D187, D188, D189, D190, D191, D192, D193, D195, D197, D198, D199, D217, D218, D219, D223, D224, D225, D226, D227, D228, D229, D255, D262, D263, D264, D266, D267, D269, D275, D276
Beef cows (*n *=* *26)	BC125, BC126, BC207, BC208, BC209, BC210, BC211, BC212, BC213, BC237, BC238, BC239, BC240, BC241, BC242, BC243, BC244, BC245, BC246, BC247, BC248, BC249, BC250, BC251, BC252, BC253
Dairy cows (*n *=* *53)	MC294 (5), MC296 (5), MC278 (4), MC289 (4), MC295 (4), MC288 (3), MC230 (2), MC231 (2), MC232 (2), MC233 (2), MC234 (2), MC235 (2), MC337 (2), MC338 (2), MC339 (2), MC345 (2), MC201, MC202, MC203, MC204, MC205, MC236, MC285, MC286, MC297, MC298, MC309, MC310, MC312, MC314, MC315, MC316, MC317, MC318, MC320, MC322, MC323, MC325, MC327, MC328, MC329, MC330, MC334, MC336, MC347, MC348, MC356, MC358, MC359, MC360, MC361, MC362, MC363

## DISCUSSION

The CRISPR-Cas system is known as an immune system in prokaryotes through the storage of spacers from foreign DNA sequences (30), which means that the presence of spacers in CRISPR loci indicates an encounter with the invasion of bacteriophages or other genetic materials. With the storage of spacers, prokaryotes logically have the potential to defend themselves against subsequent invasions from bacteriophages. Thus, the identification of spacers in CRISPR loci will help to understand the history of bacterial isolates exposed to bacteriophages or other genetic materials (31) such as mobile genetic elements, antibiotic resistance genes, and virulence genes. Thus, the documentation of a series of spacers may help develop tools for microbial source tracking, with which several studies have reported spacers of CRISPR loci in E. coli isolates from animal and human guts (26, 32–34). In this study, we characterized the spacers of CRISPR locus 2.1 and investigated their prevalence in the feces of animals and humans for the application of spacers in source tracking. The current study showed that 24.8% of the 569 E. coli isolates harbored CRISPR locus 2.1, and the occurrence of the CRISPR system was highly variable by source (beef cows, chickens, ducks, humans, dairy cows, patients, and pigs). In animals, humans, and environmental waters, 49% of E. coli strains harbor the CRISPR 2.1 regions (21). Analysis of the NCBI and CRISPRdb databases showed that CRISPR systems are not common among Klebsiella pneumoniae strains (35). Another study showed that ~37% of Klebsiella pneumoniae strains carried CRISPR systems according to complete chromosomal sequences from GenBank (36). Similarly, the occurrence rate of CRISPR systems varies among bacterial isolates. The current study showed various occurrences of CRISPR systems among E. coli isolates from animal and human sources. Thus, further investigations are required to understand the distribution of CRISPR systems in E. coli.

The current study showed that spacers of CRISPR locus 2.1 in E. coli isolates were mainly derived from *Myoviridae*, *Podoviridae*, *Siphoviridae*, and unidentified families of the *Caudovirales* order in all animal and human sources. Previous studies have reported the interactions between bacteriophages and E. coli isolates in the gut environment. CRISPR systems have been studied for bacteriophage therapy against pathogenic (13, 37) and antibiotic-resistant E. coli strains (38). Those bacteriophages have been isolated from slaughterhouse, poultry sewage, intestines of chicken and beef offal, and wastewater (15, 39–41). In addition, fecal bacteriophageome of human gut showed that the most of bacteriophage contigs identified belonged to the families of order *Caudovirales* (42). These studies indicate that most gut bacteriophageomes belong to the order *Caudovirales*, suggesting that the presence *of Caudovirales*-derived spacer sequences may indicate fecal origin. This is also likely due to the broad host range of *Caudovirales* in the closed environment of animal guts (43). However, the majority of spacers remain unidentified, because few reads from viral metagenomics of the human gut are aligned with the viral genomic reference (44).

Plasmid-derived spacers were also observed in this study and were mainly assigned to plasmids of *Enterobacteriaceae*. We found that the majority of spacer sequences were classified as plasmid pLD91-1-76kb, as previously reported in E. coli LD91-1 (45). Plasmid pLD91-1-76kb of E. coli LD91-1 was isolated from the feces of a Père David’s deer in China, carrying *mcr-1* (the mobilized colistin resistance gene). In addition, it was reported that plasmid pFORC14 in the foodborne pathogen Vibrio parahaemolyticus FORC014 was isolated from toothfish in South Korea (46). The plasmid of *Pantoea* sp. strain CCBC3-3-1 was also isolated from a Cotinus coggygria branch in China (47).

All host-identified spacers were identified in the duck E. coli CRISPR loci. We did not observe specific occurrence patterns of spacers, likely because of the lack of bacteriophage genomic data. This study, however, showed that most of the phage-derived spacers are from a few families of the order *Caudovirales*, and most of the plasmid-derived spacers are from a few genera of the *Enterobacteriaceae* family, suggesting tight associations with the intestinal environment. Notably, characterization of CRISPR spacers may provide fundamental information to track sources of E. coli: thus, investigation of these CRISPR spacers may offer a novel approach for fecal pollution source tracking. Interestingly, some spacers were specifically stored in the CRISPR arrays of E. coli from each source. The CRISPR profile of Salmonella enterica has already been proposed as an approach for source tracking (48). In addition, the CRISPR system also provides genetic evidence of the spread of antibiotic resistance genes carried by Staphylococcus (49). Analyses of the spacer profile of the CRISPR array of E. coli isolates from animals, humans, and environmental waters also suggested that a combination of methods with CRISPR analyses will prove useful in developing microbial source tracking (MST) tools (21). Accordingly, we suggest that the occurrence of source-specific spacers may help to develop a potential tool for MST.

In conclusion, we investigated the distribution of CRISPR systems and characterized CRISPR spacers within E. coli isolates obtained from animal and human feces. Our study showed that some spacers were specifically found in each source. In particular, we found that some source-specific spacers (Phaeobacter piscinae strain P13 plasmid pP13_a and phage *Inoviridae*) were bracketed in the CRISPR system of duck isolates. This suggests that more source-specific spacers could be detected by increasing the number of isolates used for CRISPR analysis. Considering the host-identified spacers, we revealed that some spacers from diverse hosts of phages and plasmids were commonly spread in the CRISPR system of E. coli isolates, and a few spacers were specifically associated with the isolates from each source. Thus, we suggest the identification of spacers in the CRISPR array of E. coli isolates as a potential approach for MST. This study could help advance further analysis of the interactions between viruses and bacteria, and MST.

## MATERIALS AND METHODS

### E. coli isolates and DNA extraction.

A total of 569 isolates of E. coli were obtained from the feces of humans and animals (50). Fecal samples from healthy humans (termed “human” in this study) were collected during annual health checkups at a hospital located in Gwangju, South Korea, in 2008. Fecal samples from human patients with diarrhea (termed “patient” in this study) were also collected at the same hospital. Genomic DNA was extracted by boiling in 0.05 N NaOH at 95°C for 15 min (17). After boiling, 1:10 dilutions of the supernatants with sterilized distilled water were immediately used as DNA templates for PCR amplification.

### Detection and sequencing of CRISPR locus 2.1.

E. coli contains two subtypes of the CRISPR system: I-E and I-F (25). The CRISPR I-E type consists of three cassettes: CRISPR 2.1, CRISPR 2.2, and CRISPR 2.3 (26). Among them, due to the highest frequency in E. coli CRISPR systems (26), CRISPR 2.1 was selected for amplification and sequencing in this study. CRISPR locus 2.1 of the E. coli isolates was amplified as previously described (51). Amplicons were visualized using a 1% agarose gel at 100 V for 15 min and captured using the Gel Doc system (Bio-Rad, USA). Variable amplicon sizes were purified using the QIAquick PCR purification kit (Qiagen, USA) and sent to Macrogen (Seoul, South Korea) for sequencing.

### Identification of spacers of CRISPR locus 2.1.

Sequences of presumptive CRISPR locus 2.1 were analyzed using CRISPRFinder (52), and protospacer and repeat sequences were manually employed and arranged in Microsoft Excel. Sequences of protospacers of CRISPR locus 2.1 were identified and predicted using CRISPRTarget (http://crispr.otago.ac.nz/CRISPRTarget/crispr_analysis.html). A cutoff score of 29 was determined as the threshold in CRISPRTarget, and the protospacers with the highest score were chosen for downstream analysis. Source-specific spacers are those present only in CRISPR arrays of one specific source among beef cows, ducks, humans, milk cows, patients, and pigs.

### Data processes.

In this study, the terms “source” and “host” indicate where E. coli isolates were obtained and where the protospacers originated, respectively. The protospacers were arranged using phages, plasmids, and an unknown source. The protospacers, identified as multiple sources, were also suspected as “unknown” in the analysis of spacer profiles. The network of spacer sources (humans and animals) and hosts was visualized using the Gephi software (53). The spacer profiles were manually visualized according to the host of the protospacers in Microsoft Excel.

### Data availability.

The sequences of CRISPR locus 2.1 in Escherichia coli isolates obtained from feces of animals and humans have been arranged by repeat and spacer sequences and can be found in Data Set S1 in the supplemental material.
